# Unhealthy food and non-alcoholic beverage advertising on children’s, youth and family free-to-air and digital television programmes in Thailand

**DOI:** 10.1186/s12889-018-5675-3

**Published:** 2018-06-15

**Authors:** Nongnuch Jaichuen, Stefanie Vandevijvere, Bridget Kelly, Vuthiphan Vongmongkol, Sirinya Phulkerd, Viroj Tangcharoensathien

**Affiliations:** 1 International Health Policy Program (IHPP), Ministry of Public Health, Nonthaburi, Thailand; 2 The University of Auckland, School of Population Health, Auckland, New Zealand; 3 University of Wollongong, Early Start Research Institute, School of Health and Society, Wollongong, Australia; 4 Institute for Population and Social Research Mahidol University Salaya, Nakhon Pathom, Thailand; 5 Health Promotion Policy Research Center (HPR), International Health Policy Program (IHPP), Ministry of Public Health, Nonthaburi, Thailand

**Keywords:** Television advertising, Food advertising and non-alcoholic beverages advertising, Children, Youth and family’s programme

## Abstract

**Background:**

Food advertising is a key factor which influences children’s food preferences. This study assessed the rates, nutritional quality and contents of food and beverage advertising in children’s, youth and family television programmes in Thailand.

**Methods:**

Free TV was recorded for two weeks in March 2014 from six to ten am and three to eight pm on weekends and three to eight pm on weekdays across all four channels; a total of 344 h recorded. Digital TV was recorded across three channels for one week for 24 h per day in October 2014; a total 504 h recorded.

**Results:**

For Free TV, 1359 food advertisements were identified, with on average 2.9 non-core food advertisements per hour per channel. The most frequently advertised food products on free TV were sugar-sweetened drinks. The rates of advertisements containing promotional characters and premium offers were significantly higher for non-core than core foods, 1.2 versus 0.03 and 0.6 versus 0.0 per hour respectively. For Digital TV, 693 food advertisements were identified, with an average of one non-core food advertisement per hour per channel. The most frequently advertised food products on digital TV were baby and toddler milk formulae.

**Conclusions:**

Food and beverage advertising on Thai television is predominantly unhealthy. Therefore, the Government and related agencies should introduce and enforce policies to address this issue. Current regulations should be adapted to control both the frequency and nature of unhealthy on-air food marketing to protect the health of Thai children.

## Background

At least 2.8 million global deaths per year are attributable to overweight or obesity [1]. Globally, the prevalence of obesity has increased two fold since 1980. In 2014, 11% of men and 15% of women aged 18 years and older were obese in Thailand, and an estimated 42 million children under five years of age were overweight in 2013 [2]. In 2014, the national health examination survey in Thailand reported that 37.5% of the Thai population aged 15 years and above were obese. The prevalence of obesity rose from 28.4% in 2009 to 32.9% 2014 among Thai men and from 40.7% in to 41.8% in the same period among women [3], while 9% of Thai children were overweight or obese in 2009 [4].

Unhealthy food advertising contributes to children’s unhealthy food choices and consumption [5–9]. The food industry in Thailand, particularly soft drink manufacturers, spends US$ 24.5 million per year on advertising their products [10]. In Thailand, 94.9% of Thai households own a television [11]. Moreover, the 2008 national mass media survey found that 99.5% of children watch television between four and eight PM and 99.9% of children watch television on weekend mornings, between four to ten AM at some moment during that period [12]. As a result, food industries advertise their products by targeting children’s television programmes [13].

Previous studies in Thailand indicated that 64% of children’s programs advertised more than 10 min per hour. Of the 39 children’s programs, 15 (38%) had advertising for non-core foods [14]. In addition those studies found that the majority of food and beverage advertising is for unhealthy products [15]. Food advertisements aired on television frequently use promotional characters (such as celebrities, athletes and cartoon characters) to attract specific audiences. Other common strategies include making the size of food larger than the actual size, emphasizing good taste, including nutrition or health claims, and creating the perception of superiority over other products [14, 15]. There is limited evidence on the rate of unhealthy food advertising targeting children in Thailand.

The National Broadcast and Telecommunications Commission issued a Notification on Criteria and Procedures for Chart List in Broadcasting B.E. 2556 (2013), which regulates the broadcasting times for children’s, youth and family programmes, where children are defined as below 15 years old and youth as below 18 years old. This Notification does not control food and non-alcoholic beverage advertising during these programmes. However, the Ministry of Public Health (MOPH) Ministerial Notification (B.E. 2550) (2007) on “Labeling of Certain Pre-cooked Ready-to-eat Food” states that ready-to-eat food and extruded snack advertisements must display text or voice messages including “Consume little and exercise for good health” [16]. This regulation does not cover sugar-sweetened drinks and unhealthy food products other than ready-to-eat foods and snacks. Other regulations also control advertising of some food products to children on television [17], such as the Notification of the Ministry of Public Health (B.E. 2550) (2007), which requires fried or baked potatoes, fried or baked corn, extruded snacks, crackers, biscuits and wafers to produce labels on packages with the following warning message: “Eat moderately and exercise for good health” using clear and bold text.

Also, advertisements of these food products on television must have a warning message with clear sound and text for at least five seconds [16]. In addition, the Food and Drug Administration Notification regarding Rules on Advertising Foods B.E.2551 (2008) states that presenters in TV advertisements, particularly for jelly, must not be medical or public health experts [18]. However, these regulations concern only labeling, and do not control the frequency of unhealthy food advertising targeting children.

This study determined the magnitude and nutritional quality of food and beverage advertising on children’s, youth and family television programmes on both free-to-air commercial television (Free TV) and Digital TV in Thailand.

## Methods

The methods of this study are based on approaches outlined in international frameworks [19] to monitor food and non-alcoholic beverages advertising on television. This work contributes to efforts to monitor food and non-alcoholic beverage promotions to children, as part of INFORMAS (International Network for Food and Obesity/Non-Communicable Diseases (NCD) research, Monitoring and Action Support), which is a global network of public-interest organisations and researchers that aims to monitor, benchmark and support public and private sector actions to create healthy food environments and reduce obesity, NCDs and their related inequalities [20].

### Population and sample

This was a nationwide study conducted across all channels (channel 3, 5, 7, MCOT) of Free TV and three channels of Digital TV (3 Family, LOCA and MCOT Family) in Thailand. All children’s, youth and family channels of Digital TV were included in our study.

### Data collection

All Free TV channels were recorded for two weeks (ten weekdays and four weekend days) from 06.00 to 10.00 am and 3.00 to 8.00 pm on weekend days (nine hours/ day/ channel) and 3.00 to 8.00 pm on weekdays (five hours/ day/ channel). A total of 344 h television of broadcasting was recorded from March 24 to April 6, 2014. The hours recorded were based on the programming times for children’s, youth and family programmes as per the Notification of the National Broadcast and Telecommunications Commission [21]. Three channels of Digital TV were recorded for a week (five weekdays and two weekend days) for 24 h per day (24 h/day/ channel) because these channel were the only children’s channels. In addition, around 3.3 million (39.9%) children aged six to fourteen years watched television on weekday evenings between eight and twelve PM and 1.6 million (19.4%) children watched television on weekend evenings between eight and twelve PM [12]. A total of 504 h digital television of broadcasting was recorded from 7 to 13 October 2014. All data were recorded from live television broadcasts onto DVDs.

### Data coding

All advertisements were coded in Microsoft Excel using the INFORMAS approach [19]. The following information was collected for each advertisement: the channel name and number, date of recording, day of the week, programme name and category, starting time of the programme, time slot of advertisement, advertisement type, company name, food product category and the presence of marketing techniques. Marketing techniques included: promotional characters (cartoons, stars, athletes, medical or public health experts, singers and celebrity endorsers) and premium offers (giveaways, competitions, contests, prize promotions, vouchers, buy one get one free and rebates). The programme category was classified into 15 types based on criteria described elsewhere [19]. All food and beverage product, supermarket and restaurant advertisements were coded into 37 food codes (See Table 3 below) that were each assigned to one of three food categories (core, non-core, and miscellaneous foods). Core foods are healthy and nutrient dense and low in discretionary energy and can be recommended to be consumed daily, while non-core foods are unhealthy and high in undesirable nutrients such as saturated fat, refined sugars, and salt [22].

Data coding was completed by three independent researchers in a two-step process. The first and second researchers coded all data independently, and any discrepancies between these two researchers were reviewed by the third researcher and resolved by consensus among the three researchers.

### Data analysis

Rates (the number of advertisements per hour, per channel, or per channel-hour) and frequencies were used to describe the food and non-food advertisements by food category, weekdays and weekend days, programme categories and their persuasive promotional techniques. Kruskal Wallis tests were used to compare the rates of food and unhealthy food advertising between channels. Post Hoc analysis for pairwise comparisons between food advertisements was carried out using Dunn test. All analyses were conducted using SPSS for Windows version 18.0 and a *p*-value less than 0.05 was considered to be significant.

## Results

On Free TV, 3240 advertisements were identified, recorded and analysed over a 14-day period (including weekend and weekdays) of which 41.9% were for foods and beverages. The overall rate of food advertising was 4.0 advertisements per channel-hour. Channel 7 had the highest rate of food advertising, at 6.2 advertisements per channel-hour on average. On Digital TV, 7838 advertisements were identified, of which 8.9% were for foods and beverages. The overall rate of food advertising was 1.4 advertisements per channel-hour. Channel 3 Family had the highest rate of food advertising, at 2.3 advertisements per hour on average (Table 1). Unhealthy non-core food advertising dominates the food adverts in all channels, with ratios ranging from one to six (Channel 5) to one to thirty-three (MCOT family).

**Table 1:** Mean rate of all and food advertisements by channel

Channel	Total ads	Mean rate of advertisements (ads per channel-hour) (SD)					Ratio core: non-core
		Total	Food	Non-core foods	Core foods	Miscellaneous foods	
Free TV	3240	9.4 (18.8)	4.0 (8.4)	2.9 (6.5)	0.2 (0.5)	0.9 (2.1)	1:14.5
3	1313	15.3 (22.3)	5.6 (8.1)	3.6 (5.5)	0.3 (0.8)	1.7 (2.6)	1:12
5	296	3.4 (8.1)	1.0 (2.9)	0.6 (1.9)	0.1 (0.5)	0.2 (0.8)	1:6
7	1109	12.9 (23.9)	6.2 (11.6)	4.7 (9.0)	0.1 (0.5)	1.4 (2.8)	1:47
MCOT	522	6.1 (14.3)	3.0 (7.8)	2.6 (6.9)	0.1 (0.3)	0.3 (1.1)	1:26
Digital TV	7838	15.6 (13.0)	1.4 (4.0)	1.0 (3.4)	0.0 (0.5)	0.4 (1.0)	1:22
3 Family	3005	17.9 (17.6)	2.3 (4.0)	1.5 (2.6)	0.1 (0.8)	0.7 (1.4)	1:15
LOCA	2343	13.9 (7.9)	0.7 (1.6)	0.4 (1.5)	0.0 (0.0)	0.3 (0.7)	N/A
MCOT Family	2490	14.8 (11.4)	1.1 (5.3)	1.0 (5.0)	0.0 (0.2)	0.1 (0.4)	1:33

Core foods are healthy and nutrient dense and recommended for daily consumption; non-core foods are unhealthy and high in undesirable nutrients such as high fat, refined sugars, and salt

### Advertising rates: difference between week and weekend days

The overall rate of food advertising on Free TV was 2.2 per channel-hour (442 advertisements) on weekdays, and 3.9 (555 advertisements) on weekend days. For all channels, with the exception of Channel 5, the rate of food advertising was highest on weekends. There were differences in Free TV advertising rates of core, non-core and other food advertisements between weekdays and weekend days. The overall rate of non-core food advertising on Digital TV was 0.8 (270 advertisements) per channel-hour on weekdays, and higher on weekend days with 1.5 (221 advertisements) per channel-hour. MCOT Family had the highest rate of food advertising on weekend days. For both week and weekend days the rates of non-core food advertising were higher than those of core food advertising on both Free and Digital TV Table 2.

**Table 2:** Mean Rate of advertising of major food groups on weekdays and weekends by TV

TV	Weekdays (ads per channel-hour (n)				Weekends (ads per channel-hour) (n)			
	Non-core	Core	Miscellaneous	*P*	Non-core	Core	Miscellaneous	*p*
Free TV	2.2 (442)^ac^	0.1 (27)^bc^	0.8 (163)^ab^	< 0.001	3.9 (555)^a^	0.2 (27)^ab^	1.0 (143)^b^	< 0.001
3	4.0^a^	0.3^ab^	1.9^b^	< 0.001	3.2	0.4	1.4	0.312
5	0.1	0.1	0.0	0.240	1.4	0.2	0.5	0.423
7	3.9^a^	0.2^ab^	1.3^b^	0.028	5.9^a^	0.1^ab^	1.4^b^	0.015
MCOT	0.9^ab^	0.0^a^	0.1^b^	0.029	4.9^ab^	0.1^a^	0.7^b^	0.006
Digital TV	0.8 (270)^ac^	0.01 (3)^bc^	0.2 (82)^ab^	< 0.001	1.5 (221)^a^	0.2 (22)^ab^	0.7 (95)^b^	< 0.001
3 Family	1.4^ac^	0.03^bc^	0.5^ab^	< 0.001	1.7^a^	0.4^ab^	1.1^b^	< 0.001
LOCA	0.5^a^	0.0^ab^	0.2^b^	< 0.001	0.2^a^	0.0^b^	0.6^ab^	< 0.001
MCOT Family	0.4^ab^	0.0^a^	0.0^b^	< 0.001	2.7^ab^	0.1^a^	0.2^b^	0.017

Values in the same row sharing same superscript letters are significantly different. n means number of food advertisements

### Programme category and core- and non-core food advertising

Free TV had the highest percentage of non-core food advertisements (73.4%; 997 advertisements), while for Digital TV this was 71% (491 advertisements).

In Free TV, the infomercial programmes [a form of TV commercial, which generally includes a toll-free telephone number or website; in Europe, this is named TV Shopping] contained 100% advertising of unhealthy non-core food items; while duringcooking programmes and children programmes 83.4 and 82% of advertisements were for non-core food items.

On Digital TV, sports programmes contained the highest proportion of food advertisements followed by documentary and infomercial programmes; where 100% of all food advertisements in these programmes were for unhealthy non-core food items. There was a greater proportion of non-core food advertisements than core food advertisements on both Free and Digital TV channels.

### Advertising rates by food product category

Free TV has higher rates of advertising of unhealthy food items - with 2.9 advertisements per channel-hour - than digital TV, which contained one advertisement per channel-hour. Advertising healthy core food items is not a priority for Free and Digital TV, with only 0.2 and 0.04 advertisements per channel-hour. (Table 3).

**Table 3:** Frequency of advertisements for food and beverage types, by Free TV

Food and beverage types	Rate of advertisements (ads per channel hour (n)	
	Free TV	Digital TV
Non-core and unhealthy food	2.9 (997)	1.0 (491)
1. High sugar and/or low fibre breakfast cereals (> 20 g sugars/100 g or < 5 g dietary fibre/100 g)	0.1	0.0
2. Flavoured/fried instant rice and noodle products	0.2	0.0
3. Sweet breads, cakes, muffins, sweet buns (e.g. lotus seed, custard, red bean), sweet biscuits (include egg rolls), sweet glutinous rice balls or cakes, high fat savoury biscuits, pies and pastries, sweet sticky rice or rice pudding.	0.1	0.0
4. Meat and meat alternatives processed or preserved in salt – include frankfurts, seafood sticks, jellyfish salad, tinned meats, and all preserved ready to eat meats, poultry, fish, tofu and egg products.	0.0	0.1
5. Sweet snack foods - include jelly, sugar-coated dried fruits or nuts, nut or seed based bars and slices, sweet rice bars, and tinned fruit in syrup	0.4	0.1
6. Savoury snack foods (added salt or fat) - includes chips, dried spicy peas, fruit chips, savoury crisps, extruded snacks, popcorn (exclude plain), salted or coated nuts, other fried snacks (e.g. shrimp crackers)	0.4	0.2
7. Fruit juice/drinks (< 98% fruit)	0.1	0.0
8. Full cream milks and yoghurts (> 3 g fat/100 g) and cheese (> 15 g fat/100 g, and high salt cheeses, including haloumi and feta) and their alternatives e.g. Soy	0.5	0.2
9. Ice cream, iced confection and desserts	0.1	0.1
10. Chocolate and candy - includes marshmallows, sugar (all types), and chewing gums (exclude sugar free varieties)	0.0	0.1
11. Fast food (not only healthier options advertised), e.g. burgers, fries, soft drinks Include if some but not all the foods/drinks advertised are healthier options	0.2	0.0
12. High fat/salt meals - frozen or packaged meals (> 6 g saturated fat/serve, > 900 mg sodium/serve). Also include steamed buns (exclude sweet buns), wontons and dumplings usually fried before consumption.	0.0	0.0
13. Other high fat/salt products– include meat/fish/bean pastes, XO sauce, butter and animal fats, high fat savoury sauces (> 10 g fat/100), soups (> 2 g fat/100 g and all dehydrated).	0.1	0.0
14. Sugar sweetened drinks - include soft drinks, sweetened tea drinks, sports/electrolyte drinks, powdered flavour additions (e.g. Nesquik, sweetened tea or coffee powders).	0.7	0.2
15. Alcohol	0.0	0.0
Core and healthy food	0.2 (56)	0.04 (25)
16. Breads, rice and rice products without added fat, sugar or salt, noodles (exclude fried), plain starch products (e.g. starch balls), plain biscuits and crackers	0.0	0.0
17. Low sugar and high fibre breakfast cereals (< 20 g sugar /100 g and > 5 g dietary fibre/100 g)	0.0	0.0
18. Fruits and fruit products without added fats, sugars or salt (include fresh, tinned in natural juice, and dried), include fruit juices containing ≥98% fruit	0.0	0.0
19. Vegetables and vegetable products without added fats, sugars or salt (include fresh, tinned, and dried), including plain seaweed	0.0	0.02
20. Milks and yoghurts (≤3 g fat/100 g), cheese (≤15 g fat/100 g) and their alternatives e.g. Soy (include probiotic drinks).	0.1	0.0
21. Meat and meat alternatives - include meat, poultry, fish, legumes, tofu, eggs and raw unsalted nuts	0.0	0.0
22. Oils high in mono- or polyunsaturated fats, (olive oil, sunflower oil, soybean oil, plant based margarines and spreads), and low fat savoury sauces (< 10 g fat/100 g).	0.0	0.0
23. Low fat/salt meals - include frozen or packaged meals (≤6 g saturated fat/serve, ≤900 mg sodium/serve), soups (< 2 g fat/100 g, exclude dehydrated), sandwiches, mixed salads. Also include steamed buns (exclude sweet buns), wontons and dumplings notusually fried before consumption.	0.0	0.0
24. Healthy Snacks – must be based on core foods (i.e. fruit, vegetables, grains, dairy, soy, meats or alternatives) and contain< 600 kJ/serve, < 3 g saturated fat/serve and < 200 mg sodium/serve	0.0	0.0
25. Baby foods (exclude milk formulae)	0.0	0.0
26. Bottled water (include unflavoured mineral and soda waters)	0.1	0.02
Miscellaneous food/food-related	0.9 (306)	0.4 (177)
27. Recipe additions (including soup cubes, oils, dried herbs and seasonings)	0.1	0.1
28. Vitamin/mineral or other dietary supplements, and sugar-free chewing gum	0.2	0.1
29. Tea and coffee (excluding sweetened powder-based teas or coffees)	0.1	0.0
30. Baby and toddler milk formulae	0.2	0.2
31. Fast food (only healthier options advertised), e.g. grilled chicken wrap, water, fruit slices	0.1	0.0
32. Fast food (only healthier options advertised), e.g. burgers, fries, soft drinks	0.1	0.0
33. Fast-food restaurant (NO foods or drinks advertised)	0.1	0.0
34. Local restaurant	0.0	0.0
35. Supermarkets (only core and healthy foods advertised)	0.0	0.0
36. Supermarkets (not only core and healthy foods advertised)	0.0	0.0
37. Supermarkets (NO foods or drinks advertised)	0.0	0.0

The top three advertised food products on Free TV were sugary drinks (17.7%, 240 advertisements), full cream milks and yoghurts (12.6%, 171 advertisements) and sweetened snack foods (11.5%, 156 advertisements); all these are unhealthy food items. The most frequently advertised product was sweetened drinks (0.7 advertisements per channel-hour).

The top three advertised food products on Digital TV were baby and toddler milk formulae (15.7%, 109 advertisements), sweetened drinks (13.7%, 95 advertisements), and savoury snack foods (11.7%, 81 advertisements). The most frequently advertised products were baby and toddler milk formulae and sweetened drinks; the rate was 0.2 advertisements per channel-hour.

### Marketing techniques

Two market strategies: use of promotional characters and premium offers are applied for analysis in Table 4. We found that the most common promotional characters were celebrity endorsers and the most common premium offers were prize promotions.

**Table 4:** Proportion and mean rate of food advertisements containing marketing techniques

Channel	Rate of promotional characters (Food ads per channel-hour) (SD)	Rate of advertisements with promotional characters (Food ads per channel-hour) (SD)			Rate of premium offers (Food ads per channel-hour) (SD)	Rate of advertisements with premium offers (Food ads per channel-hour) (SD)		
		Non-core foods	Core foods	Miscellaneous foods		Non-core foods	Core foods	Miscellaneous foods
Free TV	1.3 (3.1)	1.17 (2.7)	0.03 (0.2)	0.16 (0.5)	0.8 (2.0)	0.60 (1.5)	0.00 (0.1)	0.20 (0.7)
3	2.1 (3.2)	1.73 (2.7)	0.07 (0.3)	0.34 (0.7)	1.4 (2.4)	0.87 (1.5)	0.00 (0.0)	0.49 (1.1)
5	0.4 (1.4)	0.36 (1.2)	0.02 (0.2)	0.06 (0.3)	0.3 (1.0)	0.19 (0.7)	0.01 (0.1)	0.10 (0.3)
7	2.3 (4.5)	2.05 (4.0)	0.00 (0.0)	0.23 (0.7)	1.3 (2.5)	1.03 (2.1)	0.01 (0.1)	0.23 (0.6)
MCOT	0.6 (1.8)	0.56 (1.7)	0.01 (0.1)	0.01 (0.1)	0.4 (1.4)	0.38 (1.1)	0.00 (0.0)	0.06 (0.4)
Digital TV	0.5 (1.4)	0.45 (1.3)	0.01 (0.1)	0.04 (0.4)	0.18 (0.8)	0.16 (0.8)	0.00 (0.0)	0.02 (0.2)
3 Family	1.20 (1.9)	1.00 (1.7)	0.02 (0.3)	0.15 (0.6)	0.34 (0.8)	0.29 (0.7)	0.00 (0.0)	0.05 (0.5)
LOCA	0.09 (0.5)	0.09 (0.5)	0.00 (0.0)	0.00 (0.0)	0.01 (0.1)	0.01 (0.1)	0.00 (0.0)	0.00 (0.0)
MCOT Family	0.40 (1.4)	0.36 (1.3)	0.00 (0.0)	0.02 (0.2)	0.29 (1.1)	0.28 (1.1)	0.00 (0.0)	0.01 (0.1)

Among Free TV channels, 34.4% of food advertisements applied promotional characters. The rate was 1.3 advertisements per channel-hour, and 1.2 advertisements per channel-hour for non-core foods. Among Digital TV channels, 33.1% of food advertisements contained promotional characters. Advertisements containing promotional characters were 0.5 advertisements per channel-hour and 0.45 for non-core food items.

Most advertisements containing promotional characters and premium offers were for unhealthy non-core foods and drinks. Among Free TV channels, children were exposed to 0.6 non-core food advertisements containing premium offers every hour; while children were exposed to 0.16 non-core food advertisements containing premium offers per hour in Digital TV channels.

## Discussion

The majority of food products advertised on Free and Digital TV channels were non-core unhealthy items, with an average of 2.9 advertisements per hour per channel on Free TV and 1 advertisement per hour per channel on Digital TV. Free TV channels are more aggressive than digital TV on advertising non-core food items. The most frequently advertised foods were sugar-sweetened drinks on Free TV and baby and toddler milk formulae on Digital TV. The rate of food advertisements containing promotional characters and premium offers was higher for non-core than core foods. The rate of food and non-core food advertising was higher during weekend days than weekdays.

The dominant advertising of unhealthy food targeting children is consistent with previous studies from the Asia-Pacific region by Li D et al. [23], Kelly B et al. [22] and Ng S H et al. [24]. Free TV Channel 5 had the lowest rate of overall food advertising because the channel is owned by the Royal Thai Army, a government non-profit oriented channel. In addition, Digital TV had lower rates of food advertising than Free TV because it only started to broadcast in April 2014 [25] and has yet to attract sponsors. The majority of food advertisements on Free and Digital TV channels were unhealthy and are not yet covered by the current regulations.

In addition, regulatory instruments to restrict marketing baby and toddler milk formulae in children’s television programmes are presently limited. The National Legislative Council adopted in April 2017, a Bill on “Control of Marketing of Breast Milk Substitutes” [26]. This law, uplifting the voluntary nature of International Code of Marketing of Breast-milk Substitutes to national Act with strong enforcement, may help better control of TV advertising of this product.

There is a need to regulate the total volume and nature of unhealthy food advertising on television in Thailand. The South Korean, Special Act on Safety Management of Children’s Dietary Life includes restrictions on TV advertising of Energy-Dense and Nutrient-Poor (EDNP) foods targeting children 18 years and under. The Korean Act bans totally TV advertising of EDNP foods before, during and after all TV programmes broadcast between 5:00 and 7:00 p.m. The EDNP foods restricted by the Act included snacks and meal substitutes which were popular among children which fail to satisfy nutritional standards with threshold levels for energy, sugar, saturated fat, sodium and minimum levels of protein per single serving size set by the Korean Food and Drug Administration [27].

The non-core food advertisements contained promotional characters and premium offers. This study confirms the findings from previous studies by Ng S H et al. [24] and Kelly B et al. [28]. Kelly B et al. [29] also demonstrated that premiums and promotional characters were persuasive marketing strategies commonly used in advertisements of non-core food products in Greece, Germany, Spain, Sweden, US, Australia, UK, Canada, Italy and Brazil.

The two techniques had become a common strategy to advertise non-core food [30, 31]. Unrestricted advertising of unhealthy foods is harmful, as it creates an obesogenic environment which encourages obesity dietary behaviours among children [32]. The marketing strategies effectively influence children’s food preferences, choices and intake [33, 34].

The Fifth Ministerial Regulation B.E. 2534 (1991) issued under the Consumer Protection Act B.E. 2522 (1979) prescribes that advertisements containing premium offers shall display conditions and restrictions, such as the terms of promotion, and starting and closing dates [35]. Unfortunately, this regulation fails to control the content and form of marketing messages in TV. Moreover, the Food and Drug Administration Notification regarding Rules on Advertising Foods B.E.2551 (2008) defines that the presenters in advertising must not be from medical or public health fields. Also presenters in advertisements for instant gelatin or jelly must be older than three years old. Anyone presenting in advertisements for instant gelatin or jelly which contains glucomannan or glucomannan flour must be older than 12 years [18]. Hence, the law does not restrict using a celebrity as the presenter. These legal loopholes must be rectified to better protect the health of children.

This study found the highest rate of non-core food advertising occurred on weekends where more children are viewing TV. Similarly, research from Malaysia and the United States found that the majority of unhealthy food products advertisements was concentrated on Saturday morning programmes, and there was lower advertising of healthy food items compared to weekdays [24, 36]. In China, the five TV channels which children viewed the most had advertised unhealthy food items on weekend afternoons [23].

High rates of non-core food advertisements on weekends target children as they do not go to school and they spend leisure time watching television, on average three hour per day [37]. Additionally, the national survey found that more than 90% of children and youth are exposed to television on weekend mornings (04:01–10:00) and weekday late afternoons to evening (16:01–20:00) [12]. Moreover, available evidence indicates that TV viewing is associated with energy intake and low fruit and/or vegetable intake in children [38, 39] and contributes to childhood obesity [40].

One limitation in the design of this study is that channels of Free TV and Digital TV were not chosen to cover children’s peak viewing times since this information was not easily available, nor affordable to monitor. In addition, Free TV was not recorded over 24 h. However, the national survey confirmed that the majority of children and youth were exposed to television on weekend mornings (04:01–10:00) and weekday late afternoons to evening (16:01–20:00) and this corresponded with the hours chosen by this study [12] on Free TV channels. In addition, all advertisements were captured across all children’s, youth and family programmes. On the other hand, Digital TV was recorded for an entire week of all day and all night broadcasting. This covers all types of TV programmes and all types of advertisement in Digital TV.

## Conclusions

Free TV and Digital TV channels concentrate more on unhealthy food advertisements than on healthy or other food items. Results from this study suggest that unhealthy food and beverages occupy a majority of air space in TV advertising in both total number and rate of advertisement per channel-hours targeting children’s programmes. Though Thailand has legislated several laws and regulations to restrict marketing and advertising, the loop-holes identified by this study prompt policies to further restrict marketing and advertising of unhealthy food items that are high in saturated fats, trans-fatty acids, sugars, or salt to children. There is no specific regulation to restrict marketing and advertising for baby and toddler milk formulae targeting children’s television programmes. The government and related agencies should develop and enforce policies which restrict such practice, in particular through monitoring the enforcement of the recent Act on Control of Marketing of Breast Milk Substitutes.

## Abbreviations

EDNP: Energy-Dense and Nutrient-Poor
Free TV: free-to-air commercial television
INFORMAS: International Network for Food and Obesity/Non-Communicable Diseases (NCD) research, Monitoring and Action Support
